# Workplace mental health resilience: usability and impact study of a mental health coping mobile app on a corporate setting

**DOI:** 10.1192/j.eurpsy.2024.1149

**Published:** 2024-08-27

**Authors:** R. Maçorano, F. Canais, M. Ribas, M. Parreira, H. A. Ferreira

**Affiliations:** ^1^Neurosciences, Faculty of Sciences of the University of Lisbon, Lisbon; ^2^Psychology; ^3^Neuropsychology, NeuroGime, Braga, Portugal

## Abstract

**Introduction:**

Mental health resilience is crucial to professional wellbeing and productivity, being that 57% of company employees are reporting burnout. Additionally, early-stage preventive mental health interventions are not common, and typically employees only have access to mid-stage professional care.

**Objectives:**

The aim of this project is to provide employees with a preventive self-coping tool, enabling open and inclusive care. Specifically, the aim is to assess the receptivity, usability and impact of the usage of a mobile app that provides coping strategies based on positive psychology and a burnout-risk screening.

**Methods:**

A mobile app was used with the purpose of being accessible to everyone, independently of their financial capacity. The app also promotes inclusiveness, by aggregating several approaches and methods for mental health coping, which are recommended given the needs of each user. The app was released to a large Portuguese company with 700 employees, in which employees could download it voluntarily.

**Results:**

After 7 months, the results showed 37% receptivity rate, 24% improvement on anxiety levels, 36% improvement on workplace wellbeing, 23% increase on mental health self-coping skills, and 21% improvement on burnout-risk levels. These metrics were acquired via app’s back-end, self-reporting, and our model for burnout-risk screening.

**Conclusions:**

First results showcase the positive impact of adding such a mobile solution to the employees’ mental healthcare. Next steps will be conducting a longer study, adding control groups and productivity assessment.

**Disclosure of Interest:**

None Declared

